# Incorporating Cricket Powder into Salad Dressing: Enhancing Protein Content and Functional Attributes Through Partial Palm-Oil Replacement

**DOI:** 10.3390/foods14244268

**Published:** 2025-12-11

**Authors:** Yanjun Guo, Yu Liu, Hua Li, Chuenjit Prakitchaiwattana, Ju-Sheng Zheng, Sirithon Siriamornpun

**Affiliations:** 1 Department of Food Technology and Nutrition, Faculty of Technology, Mahasarakham University, Kantarawichai, Maha Sarakham 44150, Thailand; 2 Department of Cuisine and Nutrition, Yangzhou University, Yangzhou 225127, China; 3 Key Laboratory of Chinese Cuisine Intangible Cultural Heritage Technology Inheritance, Ministry of Culture and Tourism, Yangzhou 225127, China; 4 Department of Food Technology, Faculty of Science, Chulalongkorn University, Bangkok 10330, Thailand; 5 The Development of Foods and Food Additive from Innovative Microbial Fermentation Research Unit, Faculty of Science, Chulalongkorn University, Bangkok 10330, Thailand; 6 Westlake Laboratory of Life Sciences and Biomedicine, Hangzhou 310024, China; 7 Zhejiang Key Laboratory of Multi-Omics in Infection and Immunity, Center for Infectious Disease Research, School of Medicine, Westlake University, Hangzhou 310024, China; 8 Institute of Basic Medical Sciences, Westlake Institute for Advanced Study, Hangzhou 310024, China; 9 Research Unit of Thai Food Innovation, Department of Food Technology and Nutrition, Mahasarakham University, Kantarawichai, Maha Sarakham 44150, Thailand

**Keywords:** edible insect, E-tongue, E-nose, viscosity, sustainable ingredients

## Abstract

This study investigated the potential of cricket powder (CP) as a sustainable ingredient to partially replace palm oil in salad dressing while enhancing its functional properties. Formulations containing 0%, 5%, 7.5%, and 10% CP combined with carrageenan, guar gum, and xanthan gum were prepared. Increasing CP levels significantly decreased lightness but enhanced redness and yellowness (*p* < 0.05). Emulsion stability was significantly affected by hydrocolloid type (*p* < 0.05), with guar gum showing the highest stability, further improved at higher CP levels. Rheological analysis indicated a typical shear-thinning behavior, with xanthan gum formulations showing the highest viscosity and viscoelasticity. Moreover, CP incorporation significantly increased total phenolic content (TPC) and total flavonoid content (TFC), enhancing antioxidant activity confirmed by DPPH and FRAP assays. E-nose and E-tongue analyses revealed that increasing CP enhanced umami intensity and altered aroma profiles. Overall, replacing part of the palm oil with 5–7.5% CP improved emulsion stability and increased bioactive content, suggesting its potential as a functional and more sustainable alternative to conventional oil-rich formulations. These benefits are primarily associated with reduced palm-oil usage and increased protein and antioxidant components, rather than a fully characterized improvement in fatty-acid composition.

## 1. Introduction

In recent years, consumer interest in diverse and convenient food products has been steadily increasing. Salad dressing is an oil-in-water (O/W) emulsion product primarily composed of oil, egg yolk, vinegar, and salt [1]. It is widely appreciated by consumers for its unique flavor and versatile applications. Commercial formulations primarily rely on vegetable oils as the main lipid source, which provides desirable texture and mouthfeel [2]. Commonly used oils include soybean oil, canola oil, olive oil, and palm oil [3]. However, the high fat content of traditional salad dressing not only results in high calorie levels but is also closely associated with obesity, cardiovascular diseases, and metabolic syndrome [4]. In recent years, with the rise in global health awareness, there has been a growing demand for reduced-fat and high-protein foods [5].

Reducing or modifying the fat content in salad dressings can negatively affect texture, emulsion stability, and sensory properties [6]. To address these challenges, researchers have explored various fat replacers, including dietary fibers, proteins, and polysaccharides, to improve the texture and functional properties of fat-optimized foods [7]. In this context, partially replacing vegetable oil with cricket powder (CP) offers a promising strategy. CP is rich in high-quality proteins that can serve as natural emulsifiers, while its unsaturated fatty acids contribute to enhanced texture and nutritional value [8].

Beyond its nutritional and functional benefits, CP has gained widespread attention for its high nutritional value and environmental sustainability [9]. It contains a balanced profile of essential amino acids, including lysine, leucine, and valine, as well as amounts of unsaturated fatty acids, particularly polyunsaturated (PUFAs) and monounsaturated fatty acids (MUFAs), and bioavailable minerals such as iron, zinc, and calcium [10]. These characteristics highlight its potential as a functional ingredient to enhance the nutritional and physicochemical properties of salad dressings [11]. Furthermore, crickets require fewer resources, such as land and water, than traditional livestock, positioning them as a sustainable and eco-friendly protein source [12]. These factors make CP a viable and innovative fat replacer, offering both health benefits and environmental advantages in the development of fat-optimized, high-protein salad dressings.

Hydrocolloids play a crucial role in food systems due to their unique functional properties, as they can form gels, increase viscosity, and exhibit shear-thinning behavior. Their safety and nutritional acceptability further support their wide application in food product development [13]. Among them, carrageenan, guar gum, and xanthan gum are widely used in food emulsions but differ substantially in their mechanisms of action. Carrageenan has a well-established ability to form gels and enhance texture, contributing to better structural integrity [14]. Guar gum primarily acts as a thickening agent, enhancing viscosity and water retention without forming a true gel network [15]. Xanthan gum exhibits shear-thinning behavior, contributing to flow stability and preventing phase separation under stress conditions [16]. The distinct functional mechanisms of carrageenan, guar gum, and xanthan gum may contribute to enhanced emulsion stability in salad dressings enriched with CP. However, to date, no studies have reported on this specific application.

Despite the promising characteristics of CP, its application as a fat replacer in emulsified foods remains limited. Therefore, this study aims to evaluate the potential of CP as a partial substitute for vegetable oil in salad dressings, with the assistance of selected hydrocolloids to support emulsion stability. This work explores the feasibility of developing reduced-oil, protein-enriched, and more sustainable salad dressings while maintaining desirable texture and sensory qualities. The findings of this study could provide valuable insights into sustainable food production and contribute to the advancement of functional food innovation.

## 2. Materials and Methods

### 2.1. Materials

The CP used in this study was purchased from Protanica Co., Ltd., Samut Sakhon Province, Thailand and passed through a 60-mesh (250 µm) sieve to ensure fine particle size. The salad dressing recipe includes water, palm oil, vinegar, egg yolk, sugar, salt, and citric acid, all of which were sourced from the local market. Carrageenan (CC), guar gum (GG), and xanthan gum (XG) were purchased from Krungthepchemi (Bangkok, Thailand). All chemicals were purchased from Sigma-Aldrich Co. (St. Louis, MO, USA).

### 2.2. Salad Dressing Preparation

Salad dressing was prepared following the method of Ribes et al. [17], with modifications. All salad dressing ingredients were added to a container according to the formula proportions as shown in Table 1. The mixture was stirred at room temperature using an electric mixer at a speed of 100 rpm for 5 min. It was then heated to 80 °C and maintained for 3 min until a stable emulsion was formed. Subsequently, varying concentrations of CP (0%, 5%, 7.5%, and 10%) combined with different hydrocolloids (CC, GG, and XG) were compared with a control treatment (without CP and hydrocolloids). During the mixing process, the CP was thoroughly incorporated to ensure even distribution within the emulsion. Most hydrocolloids are typically used at concentrations below 1% in food systems; accordingly, a 1% concentration was selected to maximize functional effects while remaining within the typical usage range [13]. After mixing, the prepared salad dressing was transferred into sterilized glass containers and stored at 4 °C.

### 2.3. pH and Color

The pH and color of the dressings were determined using a pH meter and a colorimeter, respectively. The L* parameter represents brightness, ranging from 0 (black) to 100 (white). The a* coordinate denotes the red–green spectrum, where positive values indicate red tones and negative values green tones. The b* coordinate corresponds to the yellow–blue spectrum, with positive readings showing yellow shades and negative readings blue shades.

### 2.4. Emulsion Stability (ES)

The emulsion stability of the salad dressing samples was evaluated according to the method of Alpizar-Reyes et al. [18]. Approximately 30 g of sample was transferred into a 50 mL centrifuge tube and heated in a water bath at 80 °C for 30 min, and then cooled to room temperature. Next, the samples were centrifuged at 4000 rpm for 10 min, repeated three times. The remaining emulsion phase volume was measured and compared with the initial emulsion phase volume. Emulsion stability was calculated using the following formula:

(1) %ES = Final emulsion volume/Initial emulsion volume × 100

### 2.5. Rheological Properties

Rheological measurements of the salad dressing were performed on a Haake Marz 40 rheometer (Thermo Fisher Scientific, Karlsruhe, Germany) using a 35 mm parallel plate (P35/Ti), maintaining a 0.5 mm gap. The salad dressing samples were left at room temperature for 1 h before performing the rheology test.

Flow behavior measurements were conducted with shear rates from 0 to 100 s^−1^ at 25 ± 0.1 °C [19], and the rheological properties of the samples were characterized using the Power Law (2), Herschel–Bulkley (3), and Casson (4) models.

(2) τ = K_P_•(γ)^nP^

(3) τ = τ_0H_ + K_H_(γ) ^nH^

(4) τ^0.5^ = τ_0C_^0.5^ + K_C_•γ ^0.5^

where τ is shear stress (Pa), K is consistency index (Pa·s), γ is shear rate (s^−1^), and n is flow behavior index. In Equations (3) and (4), τ_0H_ (Pa) and τ_0C_ (Pa) are the Herschel–Bulkley and Casson shear stress, respectively. Equations (2)–(4), K_P_ (Pa·s), K_H_ (Pa·s), and K_C_ (Pa·s) are the Power law, Herschel–Bulkley, and Casson consistency index, respectively. Moreover, n_P_ (Pa.s) and n_H_ (Pa.s) are the Power law and Herschel–Bulkley plastic viscosities, respectively, in Equations (2) and (3).

The dynamic oscillatory test was conducted over a frequency range of 0.1–10 Hz. The storage modulus (G′), loss modulus (G″), and tan δ (G″/G′) were evaluated as functions of angular frequency for all samples under a controlled strain of 0.5% [20].

### 2.6. Microstructure

The microstructure of the salad dressing samples was observed using an optical microscope (OLYMPUS, Tokyo, Japan). After staining the samples with 1% Sudan dye, they were mounted on a glass slide and gently covered with a cover slip to ensure the surface was smooth and free of air bubbles. The samples were then observed at a magnification of 10× to assess the microstructural features of the different salad dressings.

### 2.7. Assessment of TPC, TFC, and Antioxidant Capacity

#### 2.7.1. Sample Preparation and Extraction

Extraction of the sample was performed using a method based on Jelled et al. [21], with minor modifications. Briefly, 4.0 g of the sample was extracted in 20 mL of 80% methanol at 37 °C for 12 h using an incubator shaker at 150 rpm. The mixture was subsequently filtered through Whatman No. 1 filter paper to obtain the filtrate and remove the solid residue. The resulting extract was then either analyzed immediately for the determination of total phenolic and flavonoid contents, as well as antioxidant capacity.

#### 2.7.2. Determination of Total Phenolic Content (TPC)

The TPC was measured using a slightly modified Folin–Ciocalteu method, as reported by Ratseewo et al. [22]. A 10% Folin–Ciocalteu reagent was prepared using distilled water. An aliquot of 300 µL of the extract was mixed with 2.25 mL of the reagent and allowed to react at room temperature for 5 min. Subsequently, 2.25 mL of 6% sodium carbonate solution was added and the mixture was incubated at room temperature for 90 min. Absorbance was then recorded at 725 nm with a spectrophotometer (UV-1700, Shimadzu, Tokyo, Japan) [23]. A standard curve was prepared with gallic acid (1–100 mg/L) and TPC was expressed as milligrams of gallic acid equivalents per 100 g FW (mg GAE/100 g FW).

#### 2.7.3. Assessment of Total Flavonoid Content (TFC)

TFC was measured using the colorimetric method of Siriamornpun & Kaewseejan [24], with slight modifications. Briefly, 1.0 mL of the extract was mixed with 300 µL of a 5% NaNO_2_ solution. After 5 min, 500 µL of a 2% AlCl_3_·6H_2_O solution was added and the reaction was allowed to proceed for an additional 6 min. Then, 1.0 mL of 1 M NaOH was added and the mixture was mixed thoroughly using a vortex. The absorbance was measured at 510 nm right away, with values reported as mg quercetin equivalents per 100 g fresh weight (mg QE/100 g FW).

#### 2.7.4. DPPH Free Radical Scavenging Assay

The DPPH scavenging activity of the extracts was evaluated with slight modifications to the method described by Butsat & Siriamornpun [25]. In brief, 0.5 mL of the sample extract was combined with 4.5 mL of 0.1 mM DPPH solution prepared in ethanol. After vortexing for 1 min, the mixture was left in the dark at room temperature for 30 min. Absorbance at 517 nm was measured, with results reported as milligrams of vitamin C equivalents per 100 g fresh weight (mg vitamin C/100 g FW).

#### 2.7.5. FRAP-Based Determination of Antioxidant Capacity

The FRAP method of Siriamornpun et al. [26] was applied. In the FRAP assay, 0.06 mL of extract was added to 0.18 mL deionized water was combined with 1.8 mL of freshly prepared FRAP reagent, composed of 0.3 M acetate buffer (pH 3.6), 10 mM TPTZ in 40 mM HCl, 20 mM FeCl_3_·6H_2_O, and filtered water in a 10:1:1:12 ratio at 37 °C. The mixture was incubated at 37 °C for 4 min with shaking and the absorbance was recorded at 593 nm. Results were expressed as mg FeSO_4_ equivalents per 100 g fresh weight (mg FeSO_4_/100 g FW).

### 2.8. Determination of Free Fatty Acid and Acid Value

The measurement of free fatty acids (FFAs) was performed with modifications based on the method by Gautam et al. [27]. First, 1 g of the salad dressing sample was mixed with 15 mL of ethanol and stirred well. Next, 2 drops of phenolphthalein indicator were added to the solution. The solution was titrated with 0.1 N sodium hydroxide solution until a stable pink color appeared, indicating that the endpoint of the reaction had been reached. Finally, the FFA content and acid value were calculated using the following formula:

(5) %FFA = V × N × 28.2/M

where V represents the volume of solvent used, N denotes the molarity of the NaOH solution and M is the mass of the sample.

### 2.9. E-Nose 

The volatile compounds in salad dressing were analyzed using an electronic nose (E-nose) (PEN3, AIRSENSE Analytics GmbH, Schwerin, Germany), according to the methods of Deng et al. [28] with slight modifications. The response characteristics of the 10 metal sensors are listed in Table 2. Approximately 3 g of sample was transferred into a 40 mL vial and heated in a water bath at 50 °C for 30 min. The instrumental parameters were set as follows: flush time, presampling, and measurement time were 60, 5, and 60 s, respectively. The chamber and injection flows were both set at 400 mL/minute.

### 2.10. E-Tongue 

The E-tongue (SA402B) analysis was conducted following Cai et al. [29] with slight modifications. The sensors assessed eight taste qualities: sourness, bitterness, astringency, aftertaste-B, aftertaste-A, umami, richness, and saltiness. Salad dressing samples (7 g) were mixed with 30 mL deionized water and homogenized at 10,000 rpm for 2 min. The mixture was centrifuged (10,000 rpm, 4 °C, 30 min) and the supernatant was filtered through a 0.45 μm membrane. Finally, 40 mL of the supernatant was used for measurement.

### 2.11. Statistical Analysis

Results are presented as mean ± standard deviation (SD) of three independent experiments. Prior to statistical analysis, the normality of the data was evaluated using the Shapiro–Wilk test. One-way ANOVA with Duncan’s multiple range test was used to assess statistical significance, with *p* < 0.05 considered significant (IBM SPSS Statistics 26.0). The data from the E-nose and E-tongue were analyzed using a principal components analysis (PCA) to reduce dimensionality.

## 3. Results and Discussions

### 3.1. Physical Characteristics of Salad Dressing

The physical properties of the salad dressing (SD) are shown in Table 3. For long-term storage, the pH of SD should be maintained below the recommended value of 4.0 [2]. In this study, the pH values of the samples ranged between 3.32 and 4.25, with significant differences observed between the formulations (*p* < 0.05). All formulations, except for 7.5% CC, 10% CC, 10% GG, and 10% XG, met the recommended pH level of below 4.0 for long-term storage.

The formulations containing higher levels of CP exhibited significantly lower lightness (L*) values compared with the control group (*p* < 0.05). Similar findings have been reported in previous studies, where the darker appearance was attributed to the natural brownish color of CP, which originates from the insect exoskeleton [30,31]. Significant differences (*p* < 0.05) were also observed in redness (a*) and yellowness (b*) among the formulations. Treatments with higher CP levels showed increased a* and b* values. This trend is consistent with the observations of Li et al. [32], who reported that increasing the proportion of CP in noodles led to higher redness and yellowness values. These color changes may be associated with the protein and mineral content of CP, as well as potential Maillard reactions occurring during processing, which can further influence the final color [33].

### 3.2. Emulsion Stability

Salad dressing emulsion stability (ES) is one of the most important quality parameters. The emulsion stability results of the salad dressing samples are presented in Table 3. Significant differences (*p* < 0.05) were observed among the groups, indicating that the type of hydrocolloids used had a substantial impact on emulsion stability. The control group exhibited high emulsion stability. In the samples containing carrageenan (CC), emulsion stability was relatively low, especially in CC-CP5% (46.91), which was markedly lower compared to the control (*p* < 0.05). However, increasing the CP concentration to 10% improved the stability. In contrast, samples with guar gum (GG) displayed significantly higher emulsion stability. Guar gum (GG) performed the best, possibly due to its strong thickening and gelling properties, which can form a stable network structure to restrict the movement and aggregation of oil droplets [34]. The xanthan gum (XG) groups demonstrated intermediate emulsion stability. Stability increased with higher CP concentrations, ranging from 53.42 to 61.82 (*p* < 0.05).

The observed differences in emulsion stability can be attributed to the functional properties of both the hydrocolloids and the CP. Proteins in CP may adsorb at the oil-water interface, forming a protective layer around oil droplets, while chitin and other polysaccharides present in the powder could interact with hydrocolloids to create a more structured network [35,36]. These interactions can restrict droplet movement and aggregation, increase viscosity, and enhance overall emulsion stability [37]. This may explain why samples containing guar gum exhibited the highest stability.

### 3.3. Free Fatty Acid (FFA) Content

The FFA content of salad dressings varied significantly among treatments, as shown in Table 3. The highest value was observed in the CC-CP7.5% sample (8.10%), which was significantly higher (*p* < 0.05) than most other treatments. CC-CP10% (7.55%) also showed relatively high FFA levels, while CC-CP5% (6.99%) was moderate. In contrast, samples with xanthan gum (XG-CP5%, XG-CP7.5% and XG-CP10%) exhibited the lowest FFA values, showing no significant differences (*p* > 0.05). The control (6.31%) and guar gum treatments generally fell in the mid-range, without significant differences compared to several carrageenan treatments. These results suggest that both the type of hydrocolloid and the concentration of CP influenced FFA levels in salad dressing, with carrageenan at 7.5% CP leading to the greatest increase.

The differences in FFA levels among treatments can be explained by hydrolysis and oxidation reactions. Hydrolysis is promoted by factors such as moisture, acidity, heat, and enzymatic activity, leading to the release of free fatty acids [38]. In this study, the CC-CP7.5% sample exhibited the highest FFA level (8.10%) within the same hydrocolloid group, which may be related to the local interfacial structure formed in this emulsion, making lipids more susceptible to hydrolysis. However, compared with GG and XG emulsions, CC emulsions generally showed lower stability and provided weaker protection against lipid hydrolysis, which further explains their elevated FFA levels [39].

Although the titration method does not exclusively quantify free fatty acids, as it also includes other titratable acids such as acetic and citric acid, this limitation did not affect the comparison among samples because all formulations contained identical amounts of vinegar and citric acid, ensuring a consistent baseline across formulations. Moreover, titration-based determination of acid value is a well-established analytical approach widely used in edible oil and lipid research, as demonstrated in studies on pumpkin seed oil quality, enzymatic interesterification of peanut oil, and free fatty acid reduction in crude palm oil [38,40,41]. In future work, more specific analytical techniques such as gas chromatography (GC) or ^1^H NMR will be employed to characterize individual fatty acid species and provide deeper mechanistic insight. It should be noted that the FFA content and acid value measured in this study serve as indicators of lipid hydrolysis and lipid quality, rather than representing a full nutritional characterization of the fat fraction.

### 3.4. Viscosity

**Figure 1:**
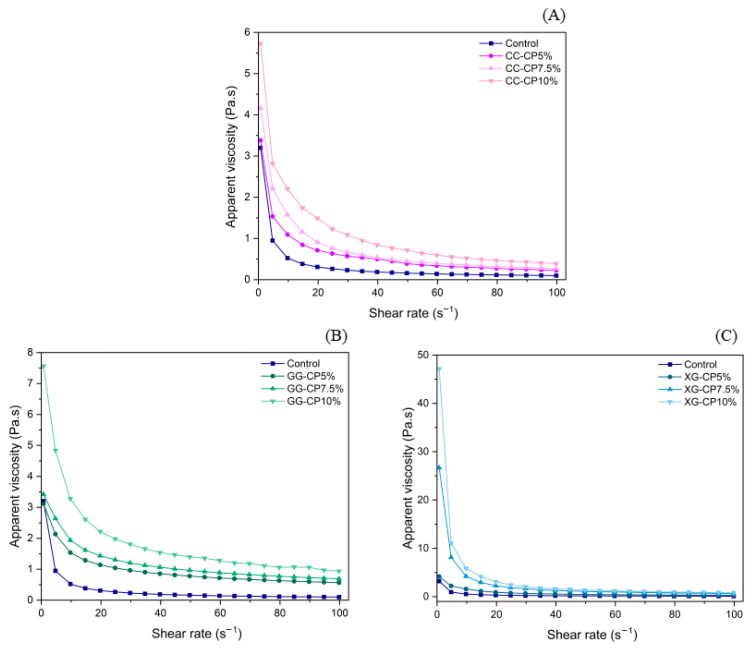
Apparent viscosity for salad dressing with different hydrocolloids: (**A**) carrageenan (CC), (**B**) guar gum (GG), and (**C**) xanthan gum (XG) containing cricket powder (CP) at 5%, 7.5%, and 10% (*w*/*w*).

Rheological properties are important characteristics of food products and play an important role in their processing [42]. Apparent viscosity versus shear rate for the salad dressing is shown in Figure 1. As the shear rate increased, the apparent viscosity of the samples decreased, indicating a shear-thinning response, which is characteristic of non-Newtonian fluids and suggests that CP-hydrocolloid dispersions exhibit pseudoplastic properties suitable for applications requiring ease of flow under shear. Among the three hydrocolloids, CC-CP samples exhibited the lowest viscosity among the hydrocolloid-treated formulations, but still higher than the control (Figure 1A), suggesting that carrageenan contributed to network formation, though its thickening ability was lower than XG and GG. GG-CP samples showed moderate viscosity enhancement with a more gradual shear-thinning effect (Figure 1B), indicating that guar gum provides structural stability through hydrogen bonding with water and proteins, leading to a smoother viscosity transition. XG-CP formulations exhibited the highest viscosity at low shear rates (Figure 1C), demonstrating xanthan gum’s superior thickening ability due to its high molecular weight and strong water-binding capacity, forming a highly structured network that resists flow [43]. The type of hydrocolloid significantly influenced the rheological properties, with XG providing the strongest viscosity enhancement, followed by GG and CC, indicating that the molecular interactions between CP and hydrocolloids play a crucial role in determining the final viscosity and flow behavior [44].

### 3.5. Analysis of Rheological Models

The rheological analysis of the samples demonstrated that all formulations exhibit shear-thinning behavior, characterized by a decrease in apparent viscosity with rising shear rate (Table 4). This non-Newtonian flow characteristic was more pronounced in samples containing hydrocolloids (CC, GG, and XG), especially at higher concentrations. The control sample showed the lowest viscosity across all shear rates, indicating a lack of structural enhancement from hydrocolloids. Among the hydrocolloids, XG-containing samples exhibited the highest viscosity, followed by GG and CC, respectively. The strong thickening effect of XG was evident, particularly at 10% concentration, where it significantly increased viscosity at low shear rates. The power-law model (K_p_, n_p_) suggests that XG-CP10% has the highest consistency coefficient (K_p_ = 37.28 Pa.sⁿ) and the lowest flow behavior index (n_p_ = 0.15), indicating strong shear-thinning properties. The Herschel–Bulkley model further confirms that hydrocolloids alter the yield stress (τ_0H_), with XG-CP10% exhibiting the highest value (τ_0H_ = 44.85 Pa), meaning it requires more force to initiate flow. Similarly, the Casson model supports this observation, as XG-containing samples display the highest yield stress and lower flow indices, suggesting stronger gel-like behavior compared to GG and CC. In terms of hydrocolloid influence, XG significantly enhances viscosity and yield stress, forming a more structured and elastic network, which contributes to a more stable system. GG provides moderate viscosity enhancement, while CC shows the least impact on viscosity but still contributes to structuring the system. The variations in rheological properties indicate that hydrocolloid type and concentration play a critical role in modulating viscosity, shear-thinning behavior, and flow characteristics, which are crucial for applications in emulsion-based food systems.

### 3.6. Frequency Sweep Tests

**Figure 2:**
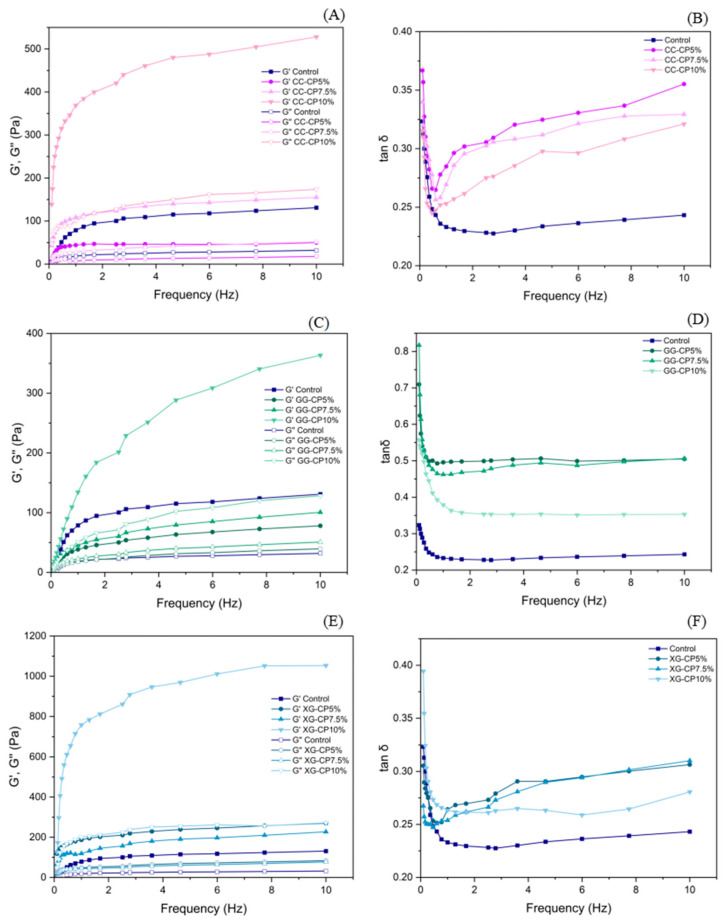
Frequency sweep tests of salad dressings formulated with (**A**,**B**) carrageenan (CC), (**C**,**D**) guar gum (GG), and (**E**,**F**) xanthan gum (XG) containing cricket powder (CP) at 5%, 7.5%, and 10%. The storage modulus (G′) and loss modulus (G″) (left panels) and tan δ values (right panels).

The frequency sweep results of salad dressings incorporated with CP and different hydrocolloids (carrageenan, guar gum, and xanthan gum) are presented in Figure 2. For all samples, G′ was greater than G″, suggesting that the dressings displayed mainly elastic, solid-like properties. An increase in frequency resulted in a noticeable rise in both G′ and G″ values, suggesting a frequency-dependent viscoelastic response. In the carrageenan-based systems (CC) shown in Figure 2A,B, the addition of CP markedly enhanced G′ and G″ compared to the control, particularly at higher concentrations (CP7.5% and CP10%). This effect suggests that CP could interact with the carrageenan network, reinforcing the gel structure and improving the elastic properties of the dressings. The tan δ values decreased with CP addition, reflecting an increase in elasticity relative to viscosity. In guar gum-based systems (GG) shown in Figure 2C,D, both G′and G″ increased progressively with the incorporation of CP. The effect was more pronounced at CP10%, where a stronger viscoelastic structure was observed. The tan δ values of GG formulations decreased with increasing levels of CP, indicating improved elastic dominance and more stable network formation. In xanthan gum-based systems (XG) shown in Figure 2E,F, the enhancement of viscoelastic moduli by CP addition was the most significant among the three hydrocolloids. G′ values, particularly at CP10%, were markedly higher than the control, suggesting synergistic interactions between xanthan gum and cricket proteins. Tan δ values were slightly reduced compared to the control, confirming greater elasticity of the matrix.

Overall, the incorporation of CP at higher levels (7.5–10%) significantly strengthened the viscoelastic structure of salad dressings, with xanthan gum formulations showing the highest moduli, followed by CC and GG. These results demonstrate that cricket CP modulates the rheological behavior of hydrocolloid-based salad dressings.

### 3.7. Microstructural Characteristics of Salad Dressings Under Microscopy

**Figure 3:**
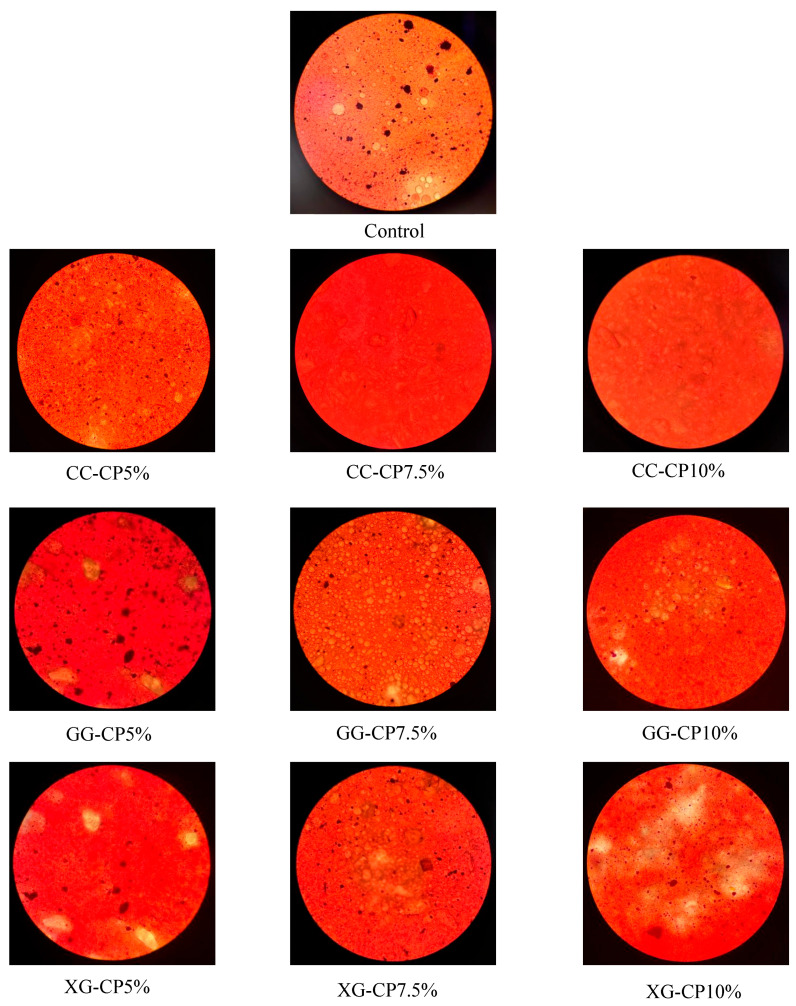
Microscopic characteristics for salad dressing with different hydrocolloids: carrageenan (CC), guar gum (GG), and xanthan gum (XG) containing cricket powder (CP) at 5%, 7.5%, and 10% (*w*/*w*).

Microscopic observations revealed distinct microstructural variations among salad dressings with different hydrocolloids (CC, GG, and XG) and CP concentrations (5%, 7.5%, and 10%) (Figure 3). CC-CP10% showed obvious particle agglomeration, with many large irregular clusters, scattered distribution and voids, resulting in poor overall dispersibility; CC-CP7.5% had a reduced degree of agglomeration compared with the 10% group, with smaller particle sizes and slightly improved distribution uniformity, but a small number of small clusters were still visible; CC-CP5% had further refined particles and a relatively stable dispersion state. GG-CP10% presented a loose flocculent structure with high porosity and loose connections between particles; GG-CP7.5% had relatively uniform particle dispersion; the XG-CP group had a poor particle dispersion effect.

The observed emulsion stability trends can be explained by these combined structural and rheological characteristics. GG-CP7.5%, which displayed uniform droplet dispersion and moderate viscosity, achieved the highest emulsion stability, likely because the balanced network structure restricted oil droplet movement without causing excessive aggregation [45]. In contrast, CC-CP10% with large aggregates had lower stability despite network formation, while XG-CP samples, although highly viscous, exhibited intermediate stability due to less uniform microstructure and potential droplet flocculation [46]. These findings highlight the interplay between hydrocolloid type, microstructure, and rheological properties in determining the functional performance of CP-enriched salad dressings.

### 3.8. Antioxidant Properties of Salad Dressing

Bioactive compounds refer to chemical agents present in natural sources that affect physiological functions via specific biological pathways. Their effects depend on the type of compound, dosage, and bioavailability [47,48]. Among these, phenolic and flavonoid compounds are well-known bioactives due to their antioxidant potential. In this study, we evaluated the total phenolics (TPC) and flavonoids (TFC) in salad dressings formulated with different hydrocolloids (Table 5). The control group exhibited the lowest TPC (37.05 mg GAE/100 g FW) and TFC (60.09 mg QE/100 g FW) values. In contrast, samples supplemented with CP and hydrocolloids showed significant improvements. In the CC treatment group, TPC increased with the concentration of CP, rising from 65.39 to 77.68 mg GAE/100 g FW. In the GG treatment group, TPC remained high across all concentrations, with the highest value reaching 80.00 mg GAE/100 g FW. For TFC, a gradual increase was observed in the CC group as the concentration of CP increased, ranging from 211.79 to 265.34 FeSO_4_/100 g FW. Similar trends in TFC were also observed in both the GG and XG groups.

The differences in TPC and TFC among the hydrocolloid-treated samples are primarily associated with the distinct microstructures formed by each hydrocolloid within the salad dressing matrix. Carrageenan may form a relatively loose and porous network, which might facilitate the diffusion of phenolic and flavonoid compounds, resulting in higher TPC and TFC values. Guar gum (GG) forms a moderately viscous and uniformly dispersed network that effectively distributes CP, leading to moderately high TPC and TFC levels. In contrast, xanthan gum (XG) produces the most compact and highly viscous structure, which may limit the release and extraction of phenolic compounds, thereby resulting in lower TPC and moderate TFC values [49].

Compared to other protein sources, such as soybean, CP samples exhibited higher TFC [50]. These results indicated that salad dressings supplemented with CP are rich in bioactive compounds, exhibiting high antioxidant activity and have the potential to be developed as functional foods [51].

Antioxidants are essential in protecting against oxidative stress, which has been shown to be closely associated with various chronic diseases, including cardiovascular diseases, cancer, and neurodegenerative disorders [52]. Therefore, evaluating the antioxidant capacity of foods is essential for the development of functional foods. In this study, two widely used methods, DPPH and FRAP assays, were conducted to evaluate the antioxidant capacity. The DPPH radical scavenging assay is a simple and commonly used method that quantifies antioxidant capacity based on the decolorization of the purple DPPH reagent [53]. Similarly, the FRAP assay evaluates the ability of samples to reduce Fe^3+^ to Fe^2+^, providing an indication of their antioxidant potential through a colorimetric change [54]. The free radical scavenging activities of salad dressings are presented in Table 5. The control group exhibited the lowest FRAP and DPPH activities, with values of 39.38 mg FeSO_4_/100 g FW and 2.75 mg vitamin C/100 g FW, respectively. The CP10%-GG and CP10%-XG groups exhibited the highest FRAP values, 81.26 and 82.17 mg FeSO_4_/100 g FW, respectively. In addition, the CP10%-GG group showed the best DPPH free radical scavenging activity, reaching 20.34 mg Vitamin C/100 g FW, significantly higher than the control group (*p* < 0.05). These results are consistent with the study by Kurdi et al. [55], which demonstrated significant DPPH free radical scavenging and FRAP activities in *B. mori*, *O. fuscidentalis*, and *G. bimaculatus*. Similarly, Anuduang et al. [56] reported strong antioxidant activity in silkworm powder, including DPPH, ABTS free radical scavenging, and FRAP values, further supporting the antioxidant potential of insect proteins. These findings suggest that the addition of CP significantly enhances antioxidant activity, likely due to its high content of phenolic compounds and flavonoids. Antioxidant properties of CP may be partly attributed to amino acids like tryptophan, cysteine, and tyrosine [57]. It is also worth mentioning that the polyphenol and flavonoid contents of edible insects may vary depending on their diet and rearing substrates, as reported in previous studies [58].

### 3.9. E-Nose

**Figure 4:**
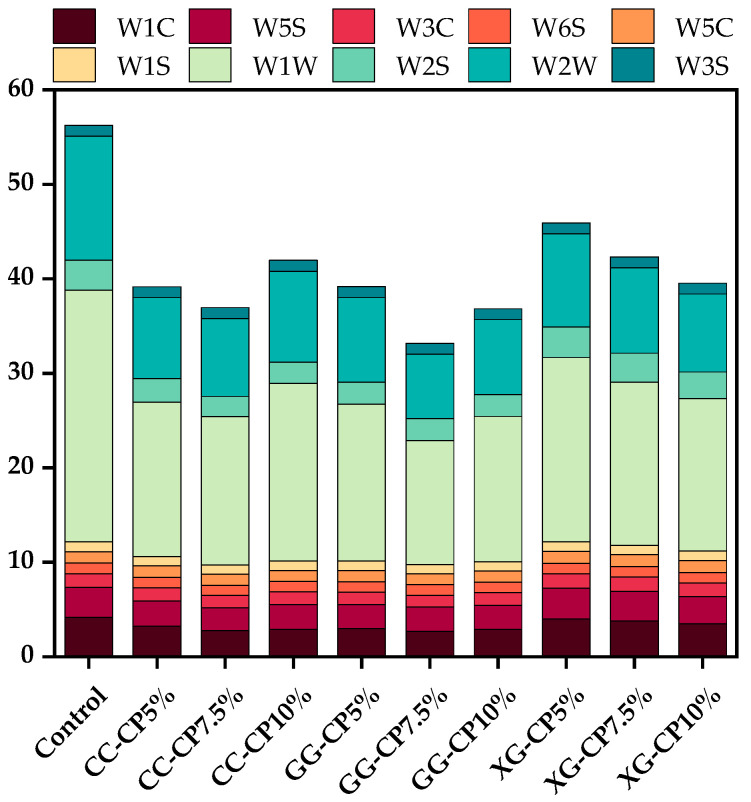
The E-nose sensor response signal values for salad dressing with different hydrocolloids: carrageenan (CC), guar gum (GG), and xanthan gum (XG) containing cricket powder (CP) at 5%, 7.5%, and 10% (*w*/*w*).

The E-nose system detects volatile substances and sample odors within a measurable range, exhibiting high sensitivity to them [59]. As shown in Figure 4, salad dressing samples had lower response values at W1C, W1W, and W2W compared to the control, indicating that there were more aromatic components, inorganic sulfides, terpenes, and organic sulfides in the control than in the other samples.

**Figure 5:**
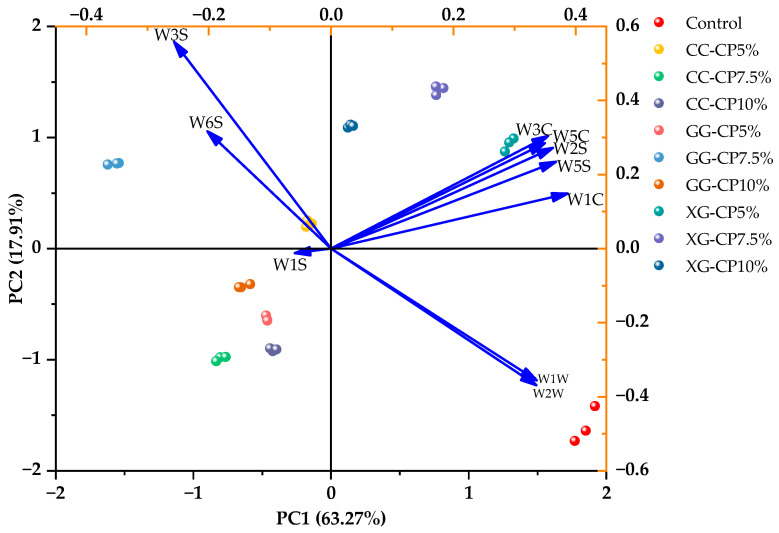
Principal component analysis score plot of the E-nose for salad dressing with different hydrocolloids: carrageenan (CC), guar gum (GG), and xanthan gum (XG) containing cricket powder (CP) at 5%, 7.5%, and 10% (*w*/*w*).

To further explore the differences in flavor profile of salad dressing with varying CP content, PCA was applied to the E-nose response data of each sample group. As illustrated in Figure 5, PC1 and PC2 explained 63.27% and 17.91% of the total variance, respectively, cumulatively explaining 81.18% of the variance. PC1 was positively correlated with W3C, W5C, W2S, W5S, W1C, W1W, and W2W, while it showed negative correlations with W1S, W6S, and W3S. Among them, W1C and W1S contributed more strongly to PC1, while W3S was more influential on PC2. The distribution of sample points correlated with distinct flavor characteristics. For example, the position of XG-CP5% was close to the arrows of W3C and W5C, indicating higher contents of aromatic and ammonia components.

In similar emulsion-based foods, such as mayonnaise, previous studies have reported that aldehydes, ketones, and alcohols produced during lipid oxidation are the major volatile compounds, and these compounds contribute significantly to flavor [60]. Therefore, in the salad dressing emulsions of this study, different formulations (e.g., varying hydrocolloid types and CP concentrations) may influence the concentrations of these volatile compounds by modulating the lipid environment (such as interfacial stability, oil–water contact, and oxidation level), which could lead to the separation of samples observed in the E-nose PCA.

### 3.10. E-Tongue 

**Figure 6:**
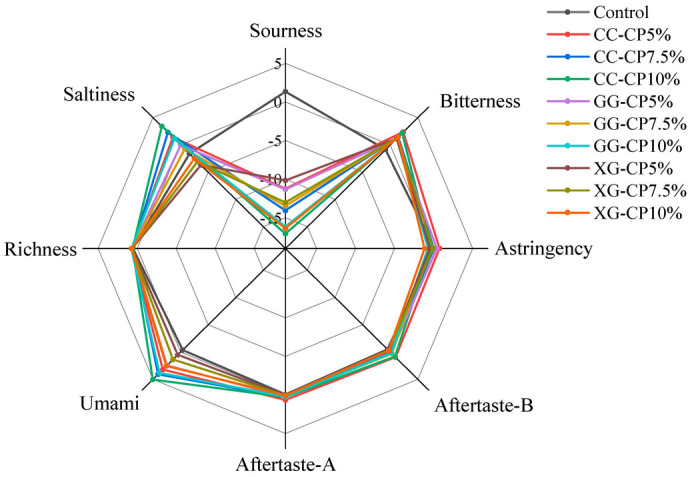
Radar chart of E-tongue sensor response signal values for salad dressing with different hydrocolloids: carrageenan (CC), guar gum (GG), and xanthan gum (XG) containing cricket powder (CP) at 5%, 7.5%, and 10% (*w*/*w*).

The electronic tongue, made up of sensors and auxiliary components, replicates human taste function while enabling non-destructive, efficient, and real-time evaluation [51]. The outcomes of the E-tongue detection are shown in Figure 6, which revealed that aftertaste-B and aftertaste-A approached zero. Furthermore, salad dressing samples with 7.5% and 10% CP exhibited no acid taste, as their taste values were below −13. A sample is considered tasteless if its taste value falls below this threshold, whereas values above the threshold indicate the presence of the corresponding flavor [61]. Salad dressing fortified with CP had higher umami than the control, and the umami increased as the percentage of CP increased. This flavor primarily arises from the thermal breakdown of proteins, producing free amino acids and enhancing the perception of umami [62].

Although the E-tongue and E-nose offer valuable objective measurements of taste and aroma profiles, these instruments cannot fully replicate human sensory perception, particularly with respect to overall acceptability, subtle flavor characteristics, and textural attributes. In the present study, the E-tongue and E-nose were utilized to obtain preliminary and quantitative assessments of sensory-related properties. Future research should integrate trained sensory panels or consumer evaluations to complement the instrumental data and provide a more comprehensive understanding of the sensory quality of cricket-powder-enriched salad dressings.

## 4. Conclusions

To explore the potential of CP as a functional ingredient in salad dressings, this study evaluated its effects on physicochemical, functional, and taste profiles measured by an electronic tongue. The incorporation of CP significantly enhanced the antioxidant capacity of the dressings, as evidenced by increases in total phenolic (TPC) and flavonoid (TFC) contents, higher DPPH and FRAP values. Rheological analysis showed that CP, particularly in combination with xanthan gum, improved viscosity and viscoelastic moduli, forming a more structured and elastic network. Emulsion stability was also enhanced, with guar gum formulations displaying the highest stability. Furthermore, electronic tongue analysis revealed that CP fortification increased umami perception without introducing undesirable aftertastes and the umami intensity increased with higher CP levels. Overall, the addition of CP not only strengthens the nutritional and antioxidant profile of salad dressings but also improves their rheological, emulsifying, and taste properties. These findings indicate that CP may serve as a natural ingredient that enhances both functional and sensory properties in partially oil-reduced emulsion-based foods. It should also be noted that the measurements of free fatty acids (FFAs) and acid value primarily reflect lipid hydrolysis and oil quality, rather than providing a complete nutritional characterization of the fat fraction. However, this study has certain limitations. The incorporation of insect powder resulted in a darker appearance of the formulations, which may influence consumer perception. Therefore, future studies should include human sensory evaluations to assess consumer acceptance of products enriched with insect-based ingredients.

## Figures and Tables

**Table 1:** Formulation of salad dressing fortified with cricket powder (%*w*/*w*).

Treatment	Palm Oil	Cricket Powder (CP)	Carrageenan (CC)	Guar Gum (GG)	Xanthan Gum (XG)	Other Ingredients
Control	32.5	0	0	0	0	Water (52.5% *w*/*w*) vinegar (10% *w*/*w*), egg yolk (3% *w*/*w*), sugar (1% *w*/*w*), salt (0.5% *w*/*w*) and citric acid (0.5% *w*/*w*)
CC-CP5%	27.5	5	1	0	0	
CC-CP7.5%	25	7.5	1	0	0	
CC-CP10%	22.5	10	1	0	0	
					
GG-CP5%	27.5	5	0	1	0	
GG-CP7.5%	25	7.5	0	1	0	
GG-CP10%	22.5	10	0	1	0	
					
XG-CP5%	27.5	5	0	0	1	
XG-CP7.5%	25	7.5	0	0	1	
XG-CP10%	22.5	10	0	0	1	

Note: Adding the difference of the sample with hydrocolloids will compensate for less water.

**Table 2:** Electronic nose sensors and their corresponding aroma type.

Sensor Number	Sensor Name	Sensitive Substance
1	W1C	Aromatic components
2	W5S	Nitrogen oxides
3	W3C	Aromatic and ammonia components
4	W6S	Hydrides
5	W5C	Aromatic components (short-chain alkanes)
6	W1S	Methyl compounds
7	W1W	Inorganic sulfides
8	W2S	Alcohols, aldehydes, and ketones
9	W2W	Aromatic components (organic sulfides)
10	W3S	Long-chain alkanes

**Table 3:** Physical characteristics of salad dressing.

Treatment	pH	Color			ES (%)	FFA (%)
		L*	a*	b*		
Control	3.32 ± 0.00 ^I^	72.84 ± 2.50 ^a^	−1.81 ± 0.30 ^f^	14.93 ± 1.67 ^d^	78.58 ± 2.90 ^a^	6.31 ± 0.51 ^cde^
CC-CP5%	3.88 ± 0.01 ^g^	60.42 ± 0.76 ^b^	2.38 ± 0.12 ^c^	16.40 ± 2.05 ^cd^	46.91 ± 0.84 ^d^	6.99 ± 0.28 ^bc^
CC-CP7.5%	4.05 ± 0.00 ^d^	56.23 ± 0.80 ^def^	3.06 ± 0.12 ^ab^	19.16 ± 0.59 ^a^	52.39 ± 0.23 ^c^	8.10 ± 0.69 ^a^
CC-CP10%	4.25 ± 0.01 ^a^	55.56 ± 0.30 ^ef^	2.88 ± 0.03 ^b^	17.29 ± 1.05 ^bc^	53.34 ± 0.38 ^c^	7.55 ± 0.08 ^ab^
GG-CP5%	3.77 ± 0.00 ^h^	60.34 ± 0.64 ^b^	2.02 ± 0.29 ^d^	18.54 ± 1.09 ^ab^	79.00 ± 3.25 ^a^	6.05 ± 0.21 ^de^
GG-CP7.5%	3.93 ± 0.01 ^f^	60.13 ± 0.26 ^b^	2.79 ± 0.07 ^b^	16.27 ± 0.01 ^cd^	82.00 ± 1.67 ^a^	6.13 ± 0.29 ^cde^
GG-CP10%	4.19 ± 0.00 ^b^	54.86 ± 0.14 ^f^	3.29 ± 0.04 ^a^	15.91 ± 0.03 ^cd^	81.94 ± 2.27 ^a^	6.86 ± 0.65 ^bcd^
XG-CP5%	3.96 ± 0.00 ^e^	58.12 ± 0.80 ^c^	1.67 ± 0.01 ^e^	11.18 ± 0.39 ^e^	53.42 ± 2.12 ^c^	5.59 ± 0.25 ^e^
XG-CP7.5%	3.95 ± 0.00 ^e^	56.65 ± 0.52 ^cde^	2.78 ± 0.16 ^b^	18.51 ± 0.08 ^ab^	59.98 ± 1.81 ^b^	5.4 ± 0.05 ^e^
XG-CP10%	4.12 ± 0.00 ^c^	57.59 ± 0.05 ^cd^	2.85 ± 0.01 ^b^	16.22 ± 0.29 ^cd^	61.82 ± 0.83 ^b^	5.42 ± 0.08 ^e^

Values are expressed as mean ± SD. Means with different letters are significantly different at *p* < 0.05 within the same column in the parameter. CC, GG, and XG are means carrageenan, guar gum, and xanthan gum. CP5%, CP7.5%, and CP10% are means of different cricket powder (5%, 7.5%, and 10%).

**Table 4:** Power law model, Herschel–Bulkley model, and Casson model of salad dressing.

Treatment	Power Law Model			Herschel–Bulckley Model				Casson Model		
	KP (Pa.s^n^)	n_P_	R^2^	τ_0H_ (Pa)	K_H_ (Pa.s)	n_H_	R^2^	*τ_C_* (Pa.s^0.5^)	*n_C_*	R^2^
Control	2.69 ± 0.05 ^j^	0.28 ± 0.01 ^de^	0.9948	2.40 ± 0.06 ^i^	0.95 ± 0.01 ^i^	0.43 ± 0.03 ^e^	0.9997	3.47 ± 0.04 ^j^	0.02 ± 0.00 ^i^	0.9927
CC-CP5%	4.03 ± 0.02 ^i^	0.42 ± 0.01 ^b^	0.9873	5.37 ± 0.02 ^h^	4.46 ± 0.03 ^f^	0.61 ± 0.02 ^c^	0.9978	6.62 ± 0.02 ^h^	0.04 ± 0.00 ^h^	0.9332
CC-CP7.5%	7.51 ± 0.04 ^g^	0.27 ± 0.01 ^e^	0.9867	14.28 ± 0.02 ^f^	15.40 ± 0.01 ^c^	0.53 ± 0.01 ^d^	0.9988	9.77 ± 0.02 ^g^	0.05 ± 0.00 ^g^	0.9538
CC-CP10%	12.21 ± 0.07 ^e^	0.29 ± 0.01 ^d^	0.9542	42.94 ± 0.01 ^c^	70.43 ± 0.07 ^a^	0.32 ± 0.01 ^g^	0.9987	15.89 ± 0.03 ^e^	0.06 ± 0.00 ^f^	0.8915
GG-CP5%	4.20 ± 0.03 ^h^	0.60 ± 0.01 ^a^	0.9998	0.64 ± 0.04 ^j^	3.65 ± 0.05 ^g^	0.63 ± 0.03 ^c^	1	5.70 ± 0.02 ^i^	0.29 ± 0.01 ^b^	0.9953
GG-CP7.5%	10.80 ± 0.10 ^f^	0.38 ± 0.01 ^c^	0.9898	5.67 ± 0.05 ^h^	7.64 ± 0.11 ^e^	0.54 ± 0.01 ^d^	0.9996	15.12 ± 0.02 ^f^	0.36 ± 0.01 ^a^	0.9898
GG-CP10%	23.98 ± 0.01 ^c^	0.30 ± 0.02 ^d^	0.9748	45.36 ± 0.03 ^a^	10.44 ± 0.10 ^d^	0.38 ± 0.01 ^f^	0.9850	30.42 ± 0.02 ^c^	0.13 ± 0.01 ^c^	0.9778
XG-CP5%	17.10 ± 0.08 ^d^	0.27 ± 0.01 ^e^	0.9736	33.90 ± 0.04 ^e^	0.01 ± 0.00 ^j^	0.98 ± 0.01 ^a^	0.9968	21.43 ± 0.03 ^d^	0.09 ± 0.00 ^d^	0.9790
XG-CP7.5%	24.82 ± 0.0 ^b^	0.21 ± 0.02 ^f^	0.9857	35.26 ± 0.07 ^d^	1.28 ± 0.05 ^h^	0.68 ± 0.01 ^b^	0.9964	30.19 ± 0.04 ^b^	0.07 ± 0.00 ^e^	0.9600
XG-CP10%	37.28 ± 0.27 ^a^	0.15 ± 0.01 ^g^	0.9458	44.85 ± 0.15 ^b^	20.94 ± 0.06 ^b^	0.16 ± 0.01 ^h^	0.9961	43.11 ± 0.02 ^a^	0.06 ± 0.00 ^f^	0.9250

Values are expressed as mean ± SD (n = 3). Means with different letters are significantly different at *p* < 0.05 within the same column in the parameter. CC, GG, and XG are means carrageenan, guar gum, and xanthan gum. CP5%, CP7.5%, and CP10% are means of different cricket powder (5%, 7.5%, and 10%).

**Table 5:** Bioactive compounds and antioxidant activity of salad dressing.

Treatment	TPC (mg GAE/ 100 g FW)	TFC (mg QE/ 100 g FW)	FRAP (mg FeSO_4_/100 g FW)	DPPH (mg Vitamin C/ 100 g FW)
Control	37.05 ± 1.68 ^i^	60.09 ± 5.72 ^f^	39.38 ± 1.52 ^e^	2.75 ± 0.36 ^g^
CC-CP5%	65.39 ± 0.13 ^f^	211.79 ± 14.25 ^b^	67.61 ± 0.20 ^c^	19.27 ± 0.05 ^d^
CC-CP7.5%	71.75 ± 0.70 ^d^	219.72 ± 9.23 ^b^	69.56 ± 0.19 ^c^	18.81 ± 0.02 ^e^
CC-CP10%	77.68 ± 0.12 ^b^	265.34 ± 0.24 ^a^	79.09 ± 0.95 ^a^	18.60 ± 0.07 ^e^
GG-CP5%	68.05 ± 0.14 ^e^	105.59 ± 0.76 ^e^	55.86 ± 0.19 ^d^	15.19 ± 0.17 ^f^
GG-CP7.5%	73.64 ± 0.34 ^c^	160.00 ± 1.61 ^d^	67.91 ± 1.84 ^c^	19.51 ± 0.07 ^cd^
GG-CP10%	80.00 ± 0.68 ^a^	205.07 ± 5.98 ^b^	81.26 ± 0.41 ^a^	20.34 ± 0.05 ^a^
XG-CP5%	34.23 ± 0.13 ^j^	160.07 ± 9.21 ^d^	70.80 ± 3.32 ^c^	18.84 ± 0.17 ^e^
XG-CP7.5%	62.02 ± 3.52 ^g^	175.40 ± 0.00 ^cd^	74.31 ± 0.82 ^b^	19.78 ± 0.02 ^bc^
XG-CP10%	58.79 ± 0.06 ^h^	181.70 ± 2.94 ^c^	82.17 ± 0.40 ^a^	19.99 ± 0.00 ^b^

Values are expressed as mean ± SD. Means with different letters are significantly different at *p* < 0.05 within the same column in the parameter. CC, GG, and XG are means carrageenan, guar gum, and xanthan gum. CP5%, CP7.5%, and CP10% are means of different cricket powder (5%, 7.5%, and 10%).

## Data Availability

The original contributions presented in this study are included in the article. Further inquiries can be directed to the corresponding author.
